# Supplementary material for: De Novo Assembly of Two Swedish Genomes Reveals Missing Segments from the Human GRCh38 Reference and Improves Variant Calling of Population-Scale Sequencing Data
Source: Genes (Basel). 2018 Oct 9;9(10):486. doi: 10.3390/genes9100486 (PMC6210158; doi:10.3390/genes9100486)
Supplement: Supplementary file 1 [file genes-09-00486-s001.zip › Supplementary_Material.pdf]

Supplementary Figures

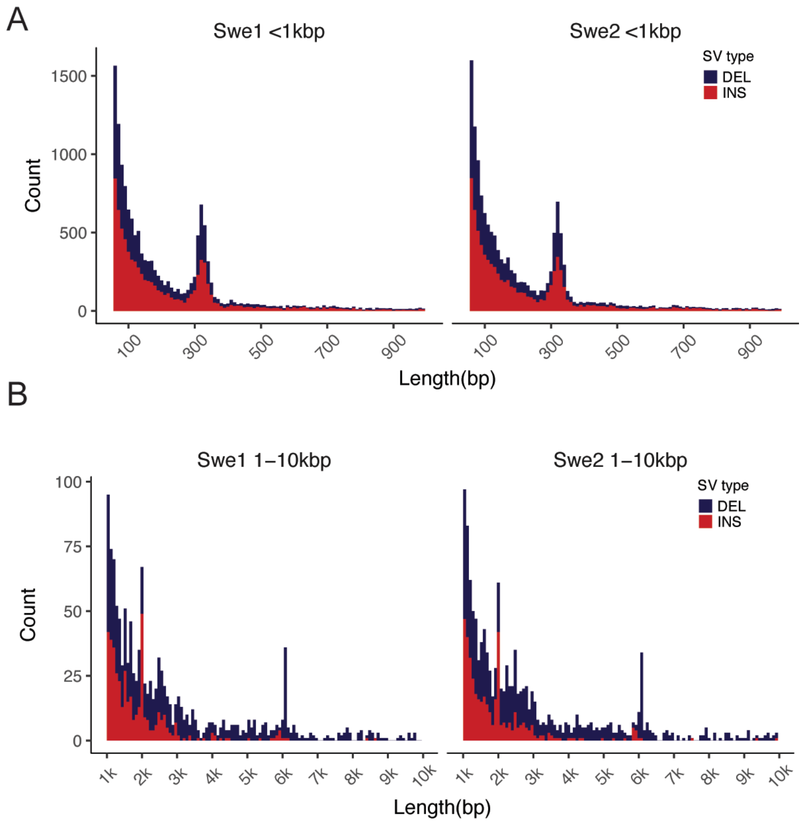

Supplementary Figure S1. Length distribution of structural variants detected in Swe1 and Swe2. **A)** Lengths of insertions (red) and deletions (blue) in Swe1 and Swe2 ranging from 50 bp to 1 kb. **A)** Lengths of insertions (red) and deletions (blue) in Swe1 and Swe2 ranging from 1 kb to 10 kb.

Supplementary Tables

Supplementary Table S1. SMRT-sequencing data overview

	Swe1	Swe2
Number of bases	236,280,502,022	233,518,501,004
Number of reads	19,676,492,492	20,319,577
N50 Read Length	16,764	15,922
Mean Read Length	12,008	11,492
Fold genome coverage[1]	78.76017	77.8395

[1]estimated for a human genome of size 3Gb

Supplementary Table S2. Results of FALCON *de novo* assembly

	Swe1	Swe2
Total contigs	7,166	7,186
Primary contigs[1]	3,139	3,162
Alternative contigs	4,027	4,024
Total bases	2,996,173,079	2,978,351,758
Total bases, primary	2,879,740,782	2,865,049,726
Total bases, alternative	116,432,297	113,302,032
N50	8,999,126	8,425,378
N50, primary	9,470,992	8,518,954
N50, alternative	30,452	29,949

[1] 20kb cut-off was used for primary contigs

Supplementary Table S3. Overview of hybrid scaffolding of PacBio data using BioNano optical maps.

		Total size	N50	Largest contig
Swe1	BspQI	3103,911 Mb	22,893 Mb	87,335 Mb
	BssSI	3044,145 Mb	26,076 Mb	127,052 Mb
	BspQI+BssSI[1]	3127,010 Mb	49,799 Mb	154,670 Mb
Swe2	BspQI	3087,923 Mb	22,914 Mb	80,150 Mb
	BssSI	3034,701 Mb	26,507 Mb	107,246 Mb
	BspQI+BssSI[1]	3103,497 Mb	45,443 Mb	107,335 Mb

[1] A two-step hybrid scaffolding was performed using first BssSI and then BspQI BioNano optical maps.

Supplementary Table S4. Alignment results of PacBio data to hg38

	**Swe1**			**Swe2**		
	Contig	Bases	Avg. Identity	Contig	Bases	Avg. Identity
Aligned	6,812 (95.06%)	2,970,533,201 (99.14%)	99.75%	6,924 (96.35%)	2,955,707,235 (99.24%)	99.74%
Unaligned	354 (4.94%)	25,639,878 (0.86%)	-	262 (3.65%)	22,644,523 (0.76%)	-
Total	7,166	2,996,173,079	-	7,186	2,978,351,758	-

Supplementary Table S5. Structural variation results for Swe1, Swe2 and HX1.

	Swe1	Swe2	HX1_50X
Type	No.	No.	No.
INS	9138	9035	7063
DEL	7342	7274	6223
DUP	1184	1081	782
INV	272	297	194
Total	17936	17687	14262

Supplementary Table S6. Statistics for NS in Swe1 and Swe2

	**Swe1**		**Swe2**	
	Sequence (>100 bp)	Base	Sequence (>100 bp)	Base
1R. mapping	3,847	21,130,809	3,964	18,478,945
2R. mapping	2,910	13,861,611	2,824	10,582,372
RemoveDuplicates	2,859	13,826,351	2,786	10,554,322

Supplementary Table S7. Repeat contents for NS is Swe1 and Swe2

	SWE1				SWE2			
	P_ctg	A_ctg	NS	NS_SWE_olp	P_ctg	A_ctg	NS	NS_SWE_olp
SINEs	13.22	12.71	2.27	1.69	13.25	12.88	2.11	1.99
LINEs	21.31	19.61	0.72	0.33	21.33	19.69	1.00	0.53
LTR	9.14	9.13	0.28	0.05	9.10	8.96	0.30	0.04
DNA	3.68	3.24	0.05	0.01	3.69	3.19	0.05	0.01
Unclassified	0.19	0.39	0.37	0.29	0.19	0.39	0.45	0.26
Satellites	1.69	2.47	42.08	49.37	1.59	2.61	37.93	39.01
Simple repeats	1.61	2.71	42.08	38.37	1.55	2.52	41.20	44.87
Low complexity	0.22	0.31	0.73	0.05	0.21	0.28	0.56	0.03
Base masked	51.08	50.62	88.58	90.17	50.96	50.57	83.60	86.74

Supplementary Table S8. GC contents for NS is Swe1 and Swe2

	SWE1	SWE2
Assembly (P20k+A)	40.95	40.95
P_ctg	40.88	40.89
A_ctg	42.67	42.51
NS	42.68	43.45
NS_SWE_overlap	44.34	43.97

Supplementary Table S9. BLAST results for NS

	**Swe1**		**Swe2**	
	Sequence	Bases	Sequence	Bases
**Eukaryota**	1,981	13,295,261	1,901	10,012,843
Human	1,759	11,414,508	1,736	9,332,030
Non-human primates	194	1,841,769	127	542,673
Mouse	8	11,884	8	10,486
Other	20	25,639	30	127,654
**N/A**	3	1,461	1	494
**Viruses**	-	-	2	24,453
**No hit**[1]	875	531,090	882	516,532
**Total**	2,859	13,826,351	2,786	10,554,322

[1] No hit includes sequences that fail to meet the 1e-50 E-value threshold.

Supplementary Table S10. Overlap of NS between Swe1, Swe2 and HX1

	**Swe1**		**Swe2**	
	Sequence	Bases	Sequence	Bases
**Shared**	678	10,553,888	491	7,330,339
with the other Swe	222	1,544,445	220	1,525,284
with HX1	113	1,361,359	44	294,295
with all	343	7,648,084	227	5,510,760
**Unique**	2,181	3,272,463	2,295	3,223,983
**Total**	2,859	13,826,351	2,786	10,554,322

Supplementary Table S11. Amount of NSs that could be anchored to hg38

	**Swe1**	**Swe2**
Anchored		
To chr1-Y (incl. alt)	2075276	1972219
To unlocalized chr	447961	443360
To unplaced scaffold	406988	280482
To multiple	1703183	1553413
To decoy	597290	732234

Decoy: EBV, bait sequences to sink reads; might be skipped

Unlocalized: sequences that are associated with a specific chr but haven't succeeded in placing on the chr (orientation and order)

Unplaced: sequences in an assembly but still cannot be associated with any chromosome

Supplementary Information

Falcon configuration file used to generate Swe1 and Swe2 assemblies

```
[General]
input_fofn = input.fofn
input_type = raw

length_cutoff = 8000
length_cutoff_pr = 8000

pa_HPCdaligner_option = -v -dal128 -t16 -e.70 -l1000 -s1000 -M28
ovlp_HPCdaligner_option = -v -dal128 -t32 -h60 -e.96 -l500 -s1000 -M28

pa_DBsplit_option = -x500 -s400
ovlp_DBsplit_option = -x500 -s400

falcon_sense_option = --output_multi --min_idt 0.70 --min_cov 4 --max_n_read
200 --n_core 8

overlap_filtering_setting = --max_diff 100 --max_cov 100 --min_cov 1 --bestn
10 --n_core 8
```
