# Supplementary figures and images for: De Novo Assembly of Two Swedish Genomes Reveals Missing Segments from the Human GRCh38 Reference and Improves Variant Calling of Population-Scale Sequencing Data
Source: Genes (Basel). 2018 Oct 9;9(10):486. doi: 10.3390/genes9100486 (PMC6210158; doi:10.3390/genes9100486)

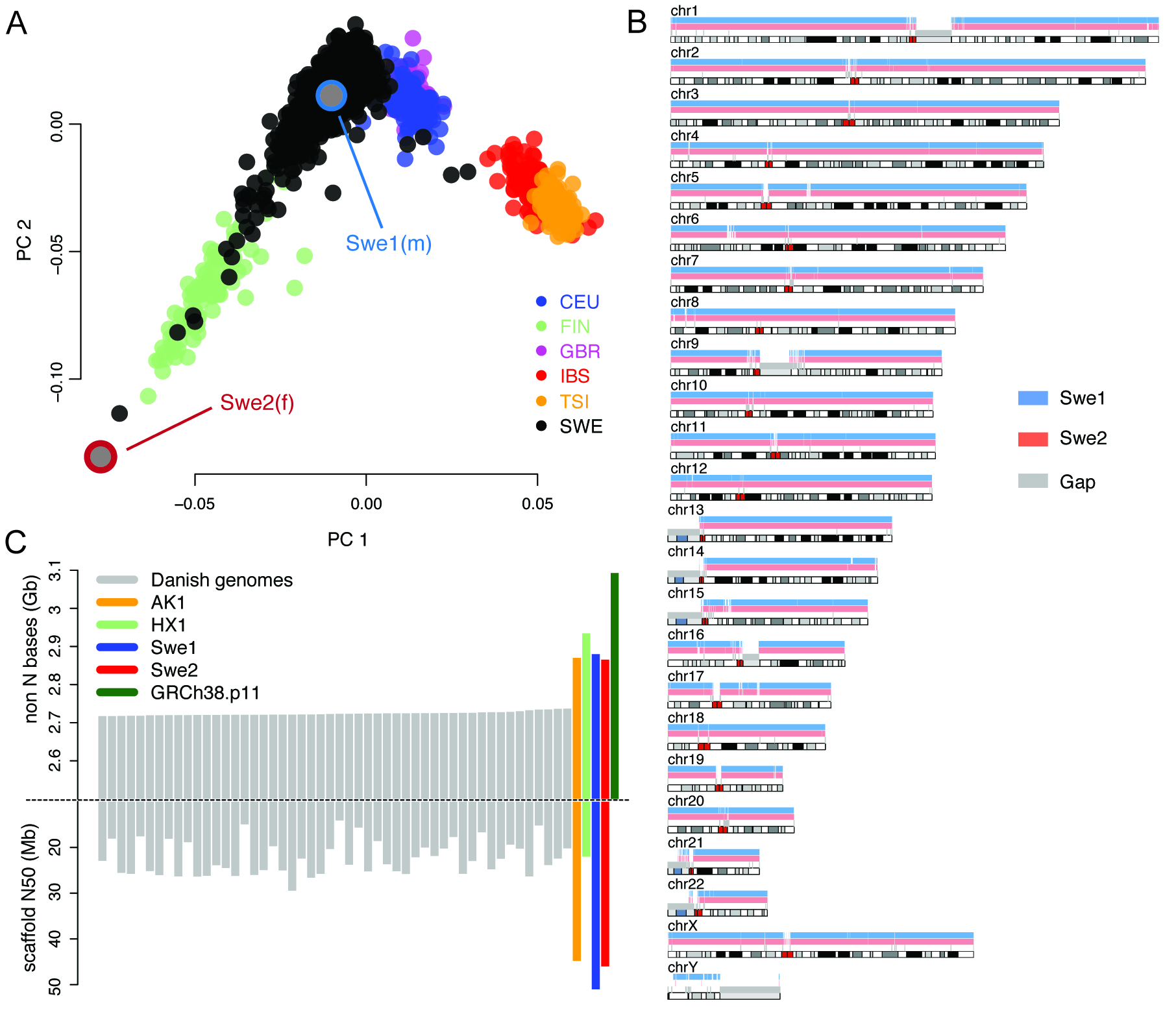

Supplement: Supplementary file 1 [file genes-09-00486-s001.zip › Figure1.tif]

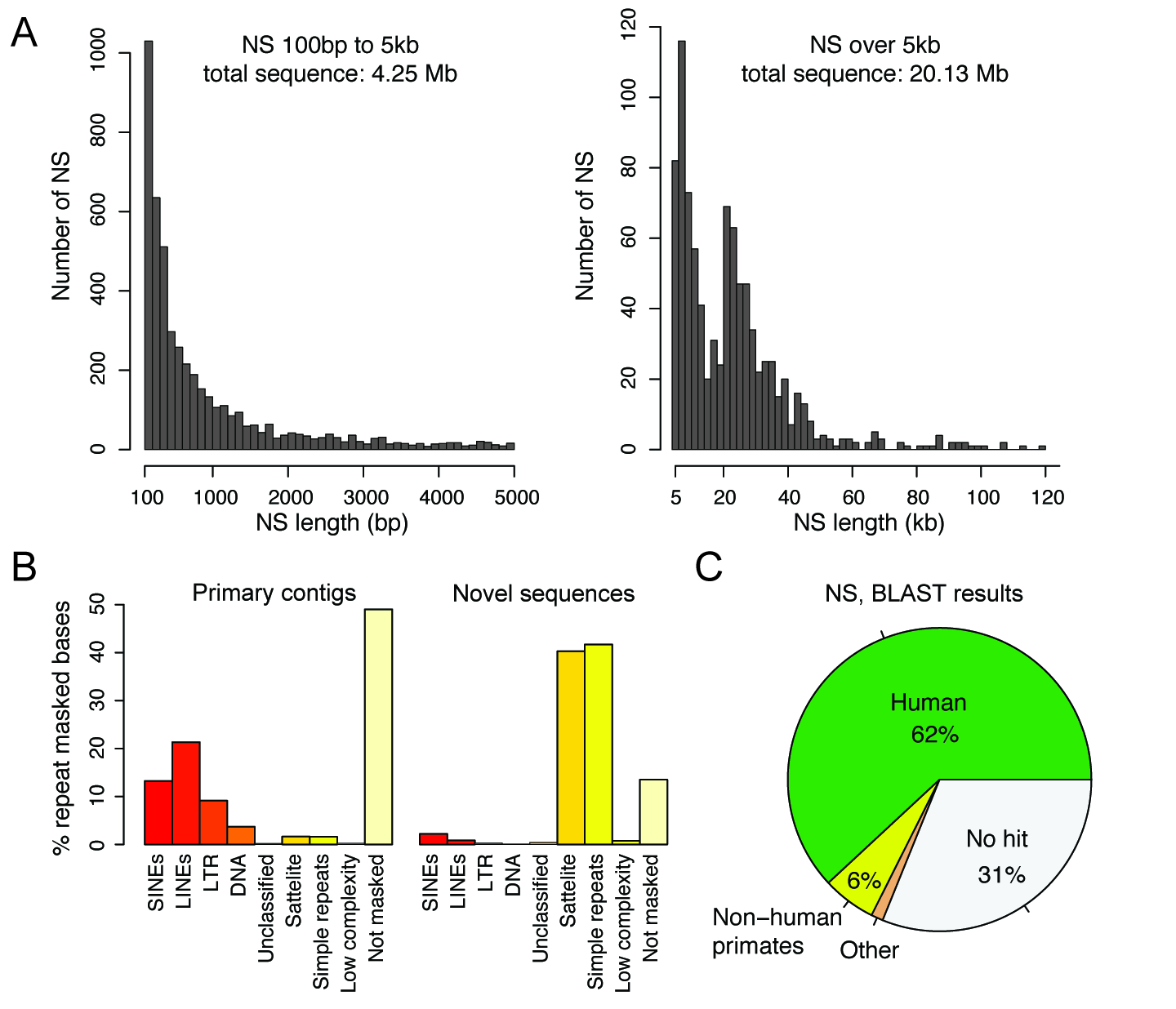

Supplement: Supplementary file 1 [file genes-09-00486-s001.zip › Figure2.tif]

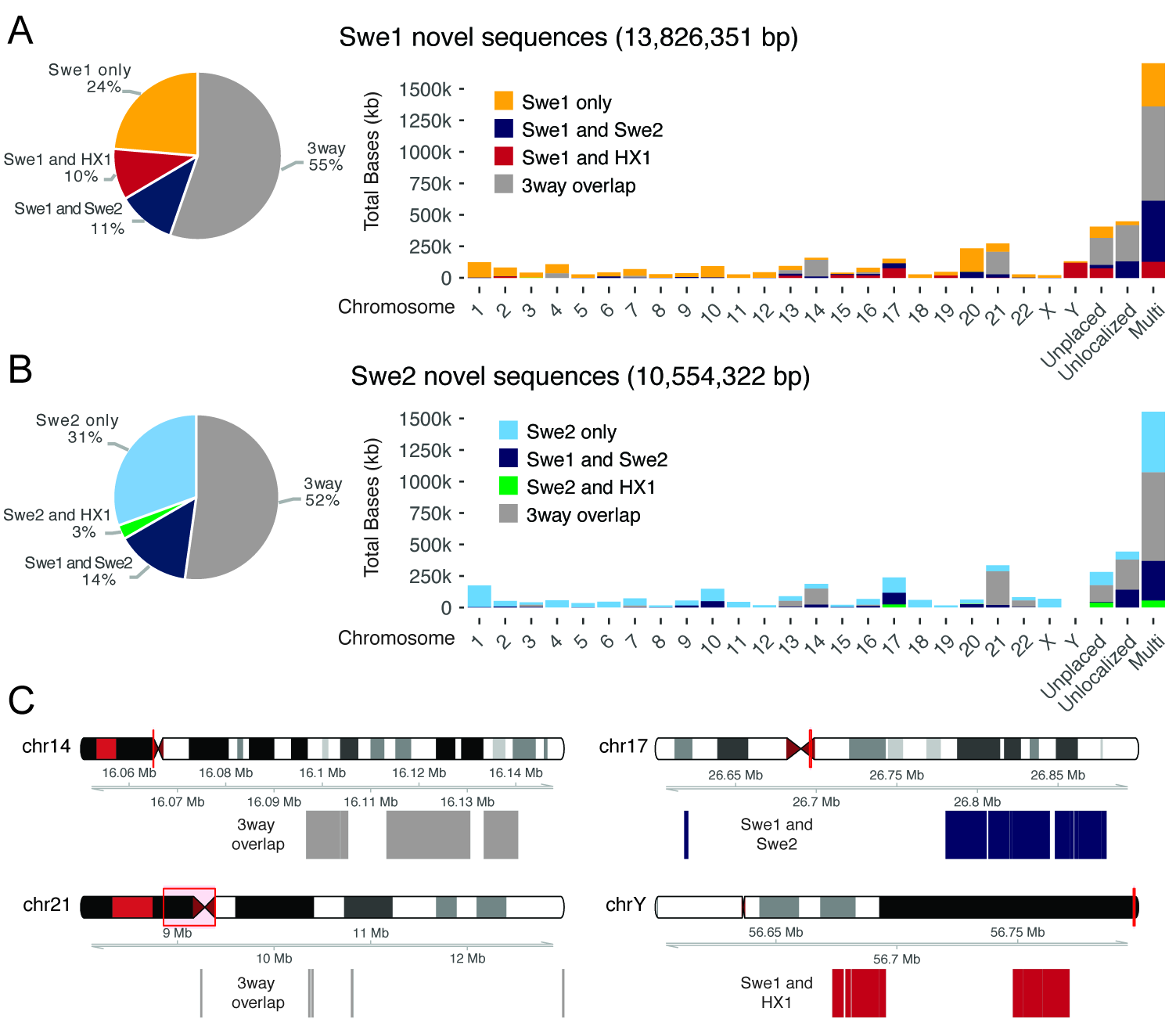

Supplement: Supplementary file 1 [file genes-09-00486-s001.zip › Figure3.tif]

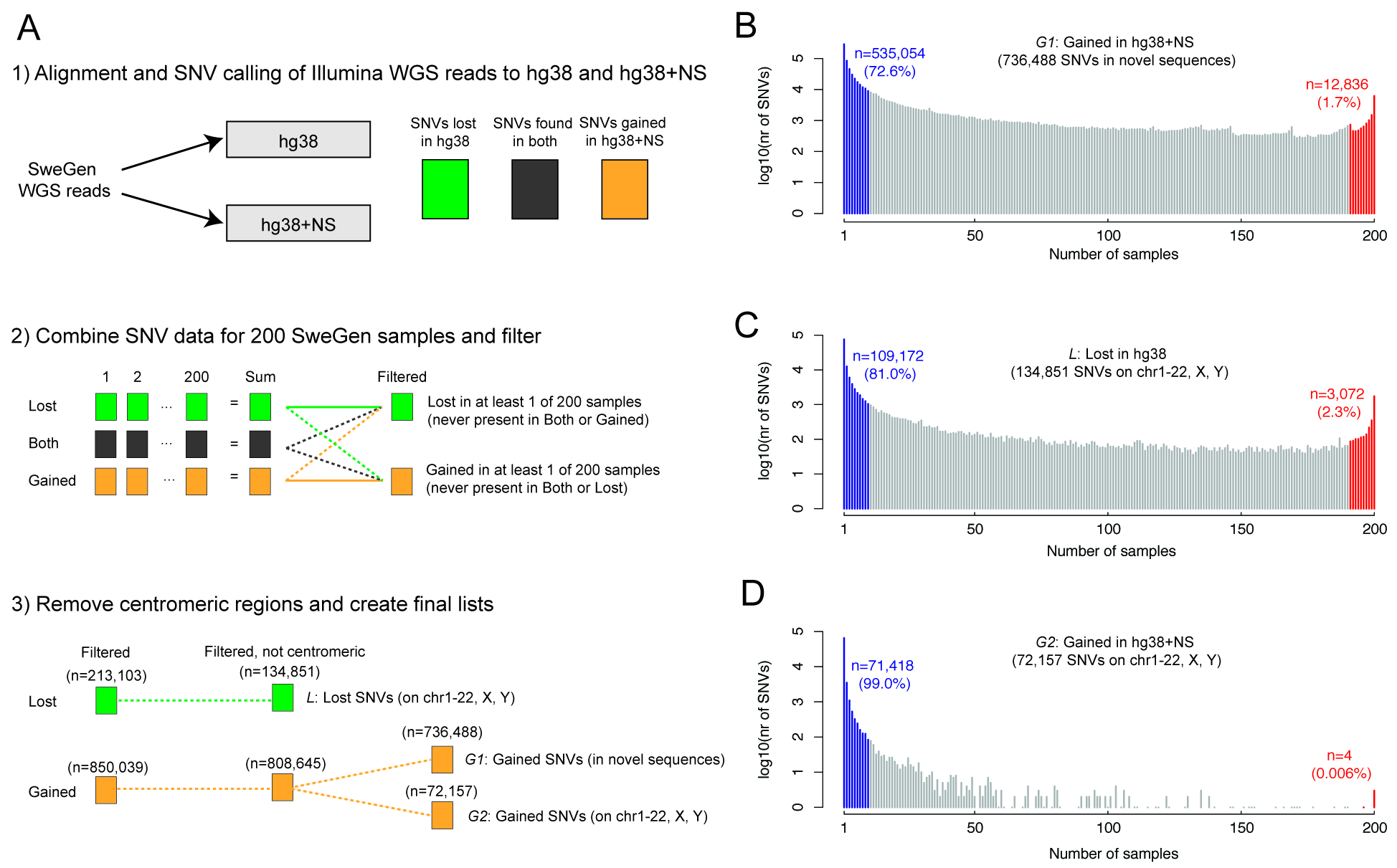

Supplement: Supplementary file 1 [file genes-09-00486-s001.zip › Figure4.tif]

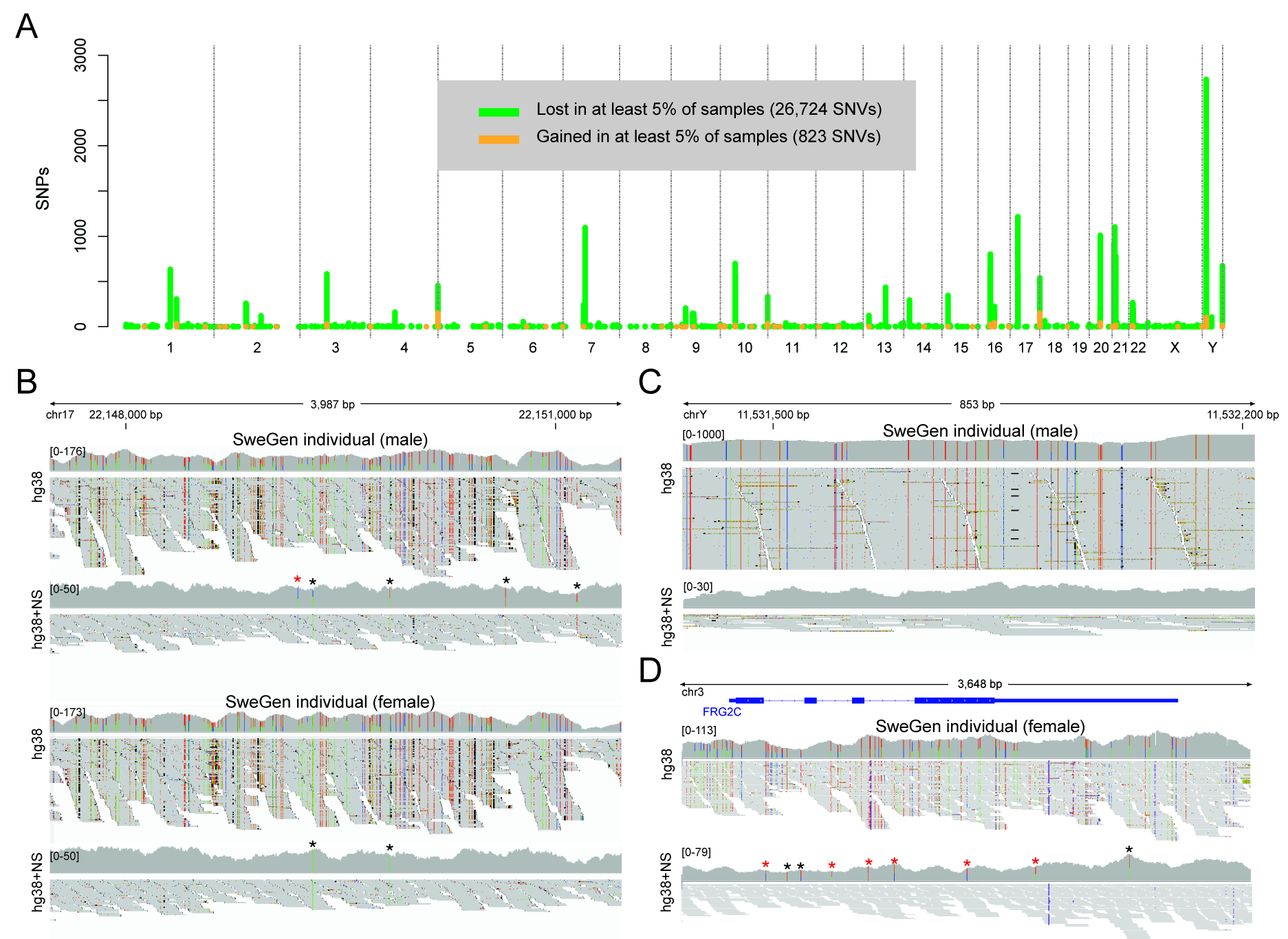

Supplement: Supplementary file 1 [file genes-09-00486-s001.zip › Figure5.tif]
